# Association Between Hormone Therapy and Health-Related Quality of Life in Postmenopausal Korean Women: A Nationwide Cross-Sectional Study Using 2005–2009 KNHANES Data

**DOI:** 10.3390/healthcare14131871

**Published:** 2026-06-26

**Authors:** Kisok Kim, Hyejin Park

**Affiliations:** 1 College of Pharmacy, Keimyung University, Daegu 42601, Republic of Korea; 2 Department of Health Sciences, Dongduk Women’s University, Seoul 02748, Republic of Korea

**Keywords:** hormone therapy, postmenopause, health status, quality of life, cross-sectional studies

## Abstract

**Background:** Hormone therapy (HT) is an effective treatment for relieving menopausal symptoms; however, its association with health-related quality of life (HRQoL) in Asian populations remains insufficiently characterized. **Objective:** The aim of this study was to examine the association between HT use and HRQoL dimensions in postmenopausal Korean women using nationally representative data from 2005, 2007, 2008, and 2009. **Methods:** In this cross-sectional study, we analyzed data from the Korea National Health and Nutrition Examination Survey (KNHANES) conducted in 2005, 2007, 2008, and 2009. Postmenopausal women aged 40–65 years were included (*n* = 2460). HRQoL was assessed using the EuroQol 5-Dimension 3-Level (EQ-5D-3L) instrument. Survey-weighted logistic regression models were used to estimate odds ratios (ORs) and 95% confidence intervals (CIs) to determine the association between HT use and each EQ-5D dimension, adjusting for age. **Results:** Of the 2460 participants, 464 (18.9%) were HT users. HT use was significantly more common among women with higher education levels (*p* < 0.001) and higher household income (*p* < 0.001). The weighted mean EQ-5D index was significantly higher among HT users (0.911, 95% CI: 0.902–0.921) than non-users (0.894, 95% CI: 0.889–0.900; *p* < 0.05). In age-stratified analyses, significant differences were observed between women aged <55 years (*p* < 0.05) and those aged ≥60 years (*p* < 0.05). The EQ-5D index was positively associated with HT duration in women aged <55 and ≥60 years (*p* for trend < 0.05). In age-adjusted, dimension-specific analyses, HT use was associated with lower odds of reporting problems across all five EQ-5D dimensions (all *p* < 0.001), with the strongest association observed for usual activities (OR = 0.719, 95% CI: 0.715–0.722). **Conclusions:** In this nationally representative sample of postmenopausal Korean women, HT use was associated with more favorable HRQoL outcomes, particularly in the usual activities domain, with patterns varying by age and BMI subgroups. These findings support individualized menopausal counseling that incorporates quality-of-life considerations into clinical decision-making.

## 1. Introduction

Menopause constitutes a critical transition in women’s lives, characterized by hormonal changes that can substantially affect health-related quality of life (HRQoL) [1]. The decline in estrogen levels during the menopausal transition is associated with a constellation of symptoms—including vasomotor disturbances, urogenital atrophy, sleep disruption, and mood changes—that collectively diminish physical, psychological, and social well-being [2]. Hormone therapy (HT), also referred to as hormone replacement therapy (HRT), has been widely used to alleviate these symptoms; however, its broader associations with HRQoL remain debated since the Women’s Health Initiative (WHI) trial raised concerns about cardiovascular and oncologic risks [3]. The publication of the WHI results in 2002 led to a dramatic decline in HT prescriptions worldwide [3]. However, subsequent re-analyses and the emergence of the “timing hypothesis” have suggested that the risks and benefits of HT may depend critically on age at initiation and time since menopause [4,5]. Specifically, HT initiated in younger women or within 10 years of menopause onset may confer cardiovascular protection rather than harm, with a more favorable overall risk–benefit profile [6]. This evolving understanding has prompted a re-evaluation of HT’s role in menopausal management, with current guidelines now endorsing individualized decision-making [7].

HT typically consists of estrogen alone (for women without a uterus) or estrogen combined with progestogen (for women with an intact uterus to prevent endometrial hyperplasia). Common formulations include conjugated equine estrogens, 17β-estradiol, and estradiol valerate, administered orally, transdermally, or through other delivery systems [8,9]. The therapeutic mechanisms of HT are multifaceted: estrogen alleviates vasomotor symptoms (hot flashes and night sweats) by stabilizing thermoregulatory centers in the hypothalamus [10,11]; improves mood and cognitive function through modulation of serotonergic and noradrenergic neurotransmission [12]; maintains bone mineral density by inhibiting osteoclast activity [13]; and preserves urogenital tissue integrity through local trophic effects [14]. Additionally, estrogen influences musculoskeletal function, cardiovascular health, and metabolic homeostasis [15,16], all of which may contribute to overall quality of life during the menopausal transition.

The EuroQol 5-Dimension (EQ-5D) instrument provides a standardized measure of health status across five dimensions: mobility, self-care, usual activities, pain/discomfort, and anxiety/depression [17]. In a previous study involving participants from the UK, Australia, and New Zealand using the EQ-5D instrument, researchers documented associations between HT and HRQoL outcomes in perimenopausal and postmenopausal women [18]. Although the association between HT use and HRQoL has been examined in studies conducted in Western countries, evidence from Asian populations remains limited, and cultural and genetic factors may differentially influence treatment outcomes [19,20]. Previous studies have yielded conflicting findings regarding HT’s effects on specific HRQoL domains. In some studies, researchers report improvements in physical functioning and emotional well-being, whereas others suggest minimal benefit or potential risk [21,22]. These inconsistencies may reflect differences in study populations, HT regimens, treatment duration, and outcome measures.

In the Korean context, the sociocultural environment surrounding menopause and HT use may differ from that of Western populations. Attitudes toward aging, patterns of healthcare utilization, and socioeconomic determinants of access to care may modify the relationship between HT and quality of life [23]. The Korea National Health and Nutrition Examination Survey (KNHANES) offers a unique opportunity to examine this association in a nationally representative sample of Korean women [24]. Clarifying the relationship between HT use and specific HRQoL dimensions may inform clinical practice and support shared decision-making between healthcare providers and patients.

This study was performed to compare HRQoL between HT users and non-users among postmenopausal Korean women, examine associations between HT use and specific EQ-5D dimensions after adjusting for age, and explore whether these associations vary by age group and sociodemographic subgroups.

## 2. Methods

### 2.1. Study Population

**Figure 1:**
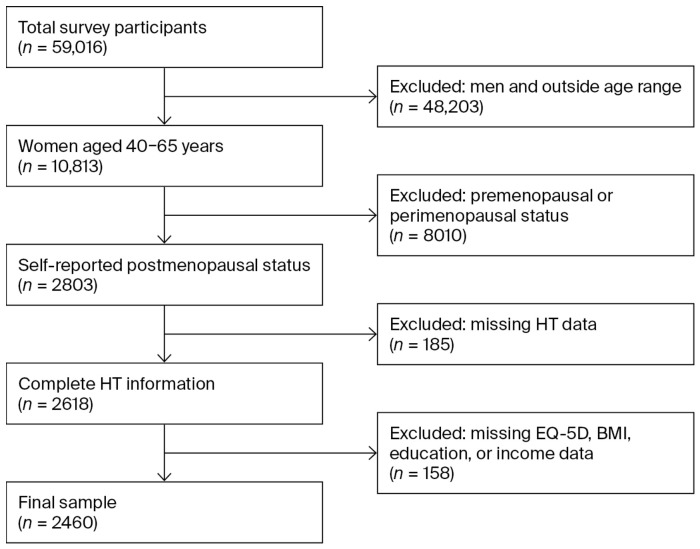
Flowchart of study participant selection from the Korea National Health and Nutrition Examination Survey (KNHANES) 2005, 2007, 2008, and 2009. HT = hormone therapy; EQ-5D = EuroQol 5-Dimension. The initial KNHANES sample included all survey participants across the four survey cycles. Women were excluded if they were aged outside the 40–65 year range, had premenopausal status, had missing data on key variables (height, weight, household income, or education level), had incomplete EQ-5D responses, or had missing HT status or duration information. The final analytical sample comprised 2460 postmenopausal women (HT users: *n* = 464; non-users: *n* = 1996).

In this cross-sectional study, we used data from the KNHANES, a nationally representative survey conducted by the Korea Centers for Disease Control and Prevention. We analyzed combined data from the 2005, 2007, 2008, and 2009 survey cycles because these cycles represent the only period during which both comprehensive HT usage data and standardized HRQoL assessment using the EQ-5D-3L instrument were concurrently collected in postmenopausal women. The KNHANES employs a stratified, multistage, clustered probability sampling design to represent the non-institutionalized civilian population of South Korea [24]. The inclusion criteria were female sex, age of 40–65 years, and confirmed postmenopausal status. Postmenopausal status was defined based on survey-specific questions. Exclusion criteria included missing data on key variables (age, sex, height, weight, income, education), premenopausal status, incomplete EQ-5D responses, and missing HT status or duration (among users). The final analytical sample comprised 2460 postmenopausal women (Figure 1).

### 2.2. HT Assessment

Current HT use was ascertained through standardized questionnaires. Participants reporting current or past use were classified as HT users, whereas those who had never used HT were classified as non-users. Among HT users, duration of use was categorized as follows: 0 months (non-use), >0–12 months (short-term), and >12 months (long-term). Treatment duration was dichotomized as short-term vs. long-term based on clinical and biological rationale. The 12-month threshold aligns with (1) the typical timeframe for maximal vasomotor symptom relief; (2) durations commonly recommended in clinical guidance for evaluating therapeutic benefit before considering discontinuation; and (3) the time required for measurable changes in bone mineral density and musculoskeletal outcomes [25,26,27]. This categorization also ensured adequate sample size in both groups for stratified analyses. Participants with missing duration data were excluded from duration-specific analyses.

### 2.3. HRQoL Assessment

HRQoL was measured using the Korean version of the EQ-5D 3-Level (EQ-5D-3L) instrument. The EQ-5D-3L assesses health status across five dimensions: mobility, self-care, usual activities, pain/discomfort, and anxiety/depression. Each dimension is rated on a three-point scale: no problems (level 1), some problems (level 2), or extreme problems (level 3). For logistic regression analyses, responses were dichotomized as no problems (level 1) versus any problems (levels 2–3). The EQ-5D index score was calculated using Korean-specific value weights developed by the Korea Centers for Disease Control and Prevention [28].

### 2.4. Covariates

Demographic and socioeconomic covariates included age, categorized as <55, 55–59, and ≥60 years; body mass index (BMI), categorized as <18.5 kg/m^2^ (underweight), 18.5–22.9 kg/m^2^ (normal), 23.0–24.9 kg/m^2^ (overweight), and ≥25.0 kg/m^2^ (obese); education, categorized as ≤elementary school, middle school, and ≥high school; and household income, categorized by quartiles (Quartile 1 [lowest] to Quartile 4 [highest]).

### 2.5. Statistical Analysis

All analyses incorporated KNHANES weights, strata, and primary sampling units to account for the complex survey design and to provide nationally representative estimates. Descriptive statistics included weighted means with 95% confidence intervals (CIs) for continuous variables and weighted frequencies for categorical variables. Between-group differences in baseline characteristics were assessed using the Mantel–Haenszel chi-square test. Differences in mean EQ-5D index scores between HT users and non-users were evaluated using weighted *t*-tests. Survey-weighted logistic regression models were used to estimate odds ratios (ORs) and 95% CIs for the association between HT use and each EQ-5D dimension, adjusting for age. Trends in EQ-5D index scores across HT duration categories (0, >0–12, and >12 months) were assessed using a *p*-for-trend test within each age stratum. Statistical significance was defined as *p* < 0.05 (two-tailed). All analyses were conducted using SAS version 9.4 (SAS Institute Inc., Cary, NC, USA).

### 2.6. Ethical Considerations

The KNHANES was conducted with approval from the Institutional Review Board of the Korea Centers for Disease Control and Prevention. All participants provided written informed consent. This secondary analysis was conducted in accordance with the Declaration of Helsinki and relevant ethical guidelines.

## 3. Results

### 3.1. Study Population Characteristics

The final analytical sample included 2460 postmenopausal women (Table 1). The prevalence of HT use was 18.9% (*n* = 464) and exhibited a decreasing trend with advancing age, although this difference did not reach statistical significance (*p* = 0.082). Among women aged <55 years, 20.4% were HT users, compared with 19.3% among those aged 55–59 years and 17.2% among those aged ≥60 years. HT use was significantly associated with BMI (*p* = 0.010), with the highest prevalence observed among normal-weight women (22.7%). Women with higher education levels were also significantly more likely to use HT (25.9% among those with ≥high school education, *p* < 0.001). Similarly, HT use was most prevalent in the highest household income quartile (25.3%, *p* < 0.001).

### 3.2. HRQoL by HT Status

The weighted mean EQ-5D index with 95% CIs by demographic characteristics, stratified by HT status, is presented in Table 2. The overall weighted mean EQ-5D index was 0.898 (95% CI: 0.893–0.903), with HT users exhibiting a significantly higher score (0.911, 95% CI: 0.902–0.921) than non-users (0.894, 95% CI: 0.889–0.900; *p* < 0.05). In age-stratified analyses, HT users aged <55 years exhibited a significantly higher mean EQ-5D index (0.939, 95% CI: 0.925–0.952) than non-users (0.919, 95% CI: 0.911–0.927; *p* < 0.05). In the 55–59-year age group, no significant difference was observed between HT users (0.892, 95% CI: 0.873–0.910) and non-users (0.906, 95% CI: 0.896–0.917). Among women aged ≥60 years, HT users again exhibited significantly higher EQ-5D scores (0.895, 95% CI: 0.877–0.913) than non-users (0.857, 95% CI: 0.849–0.868; *p* < 0.05). With respect to BMI, only women in the highest BMI group (≥25.0 kg/m^2^) demonstrated a significant difference: HT users exhibited higher EQ-5D scores (0.900, 95% CI: 0.882–0.918) than non-users (0.871, 95% CI: 0.861–0.881; *p* < 0.05). No significant differences were observed in the lower BMI groups. In analyses stratified by education level, HT users tended to exhibit higher EQ-5D scores across education categories; however, these differences did not reach statistical significance. Similarly, differences between HT users and non-users within each household income stratum were not statistically significant.

### 3.3. ORs for EQ-5D Dimensions

The age-adjusted ORs for reporting any problems in each EQ-5D dimension by HT status are presented in Table 3. HT use was associated with significantly lower odds of reporting problems across all five dimensions. The strongest association was observed for usual activities (OR = 0.719, 95% CI: 0.715–0.722), followed by self-care (OR = 0.855, 95% CI: 0.848–0.862) and mobility (OR = 0.869, 95% CI: 0.865–0.872). Anxiety/depression exhibited an intermediate association (OR = 0.915, 95% CI: 0.911–0.918), whereas pain/discomfort demonstrated the most modest association (OR = 0.990, 95% CI: 0.987–0.993). All associations were statistically significant (all *p* < 0.001).

In age-stratified analyses, associations between HT use and reporting problems in each EQ-5D domain differed across age groups (Table 4). Among women aged 40–54 years, HT use was significantly associated with lower odds of reporting problems across all EQ-5D domains (all *p* < 0.001). Similarly, among those aged 60–65 years, HT use was associated with reduced odds of reporting problems in mobility, self-care, usual activities, and pain/discomfort (all *p* < 0.001); in comparison, no significant association was observed for anxiety/depression (OR = 1.005, 95% CI: 0.998–1.012; *p* = 0.174). In contrast, among women aged 55–59 years, HT use was significantly associated with increased odds of reporting problems across all EQ-5D domains (all *p* < 0.001).

### 3.4. EQ-5D Index by HT Duration and Age Group

The mean EQ-5D index according to HT duration, stratified by age group, is presented in Table 2. HT duration was classified into three categories: no HT use (0 months), short-term use (>0–12 months), and long-term use (>12 months). Longer HT duration was significantly associated with higher EQ-5D index scores among women aged <55 years and those aged 60–65 years (*p* for trend < 0.05). However, among women aged 55–59 years, no significant association between HT duration and EQ-5D index scores was observed.

## 4. Discussion

The results of this nationally representative study of 2460 postmenopausal Korean women demonstrate significant associations between HT and HRQoL. The overall weighted mean EQ-5D index was modestly but significantly higher among HT users (0.911, 95% CI: 0.902–0.921) than non-users (0.894, 95% CI: 0.889–0.900; *p* < 0.05). Although the absolute difference appears small, at the population level, even modest differences in utility scores may translate into meaningful differences in quality-adjusted life years and may have implications for health economic evaluations [29]. A key finding was the age-specific pattern of associations. A significant difference in EQ-5D scores between HT users and non-users was observed among women aged <55 years (0.939 vs. 0.919; *p* < 0.05) and among those aged ≥60 years (0.895 vs. 0.857; *p* < 0.05), with no significant difference observed in the 55–59-year age group. The finding in younger women is consistent with the “timing hypothesis”, which posits that HT initiated closer to menopause onset may confer greater benefit [5,6]. The association observed in the ≥60-year age group may reflect longer-term HT use among women who initiated therapy earlier and continued treatment; however, the cross-sectional design precludes definitive causal inference.

Dimension-specific analyses revealed variation in the magnitude of association across EQ-5D domains. In the age-adjusted model, HT use was associated with lower odds of reporting problems in all five dimensions, with the strongest association observed for usual activities (OR = 0.719), followed by self-care (OR = 0.855) and mobility (OR = 0.869). The relatively strong association for usual activities, representing a 28% lower odds of reporting problems, suggests that HT may particularly benefit functional capacity in daily life. However, when interpreting the findings, one should consider that HT users in this sample were more highly educated, had higher household income, and had lower BMI, raising the possibility of a “healthy user” effect in addition to potential therapeutic effects. Several biological mechanisms may underlie the relatively strong association between HT use and usual activities. Estrogen influences muscle mass, bone density, and joint function, which may help preserve physical capacity for daily tasks [30]. In addition, estrogen modulates pain perception through effects on nociceptive pathways and inflammatory processes, potentially supporting continued engagement in usual activities despite other health challenges. The comparatively modest association observed for pain/discomfort (OR = 0.990) relative to usual activities (OR = 0.719) suggests that any functional benefit may extend beyond pain reduction alone.

The BMI-stratified analysis demonstrated that, among women with a BMI of ≥25.0 kg/m^2^, HT users exhibited significantly higher EQ-5D scores than non-users (0.900 vs. 0.871; *p* < 0.05), whereas no significant differences were observed in lower BMI groups. This pattern suggests that women with obesity may derive particular HRQoL benefit from HT. Obesity is associated with greater menopausal symptom burden and may exacerbate the adverse effects of estrogen deficiency on musculoskeletal function and overall quality of life [31]. Conversely, obesity may alleviate certain menopausal symptoms by compensating for the marked decrease in ovarian estrogen and becoming the primary source of estrogen through aromatase-mediated conversion of adrenal androgens [32,33]. The degree of postmenopausal estrogenization is therefore largely determined by adiposity, as adipose aromatase activity increases with body mass and age [34]. The results of a recent study revealed depot-specific differences in estrogen metabolism, with visceral adipose tissue exhibiting higher estrone concentrations and increased estrone-to-estradiol conversion that correlates with waist circumference [35]. Furthermore, menopausal HT significantly modulates intracrine sex steroid metabolism in adipose tissue, with HT users demonstrating higher local estrogen-to-androgen ratios and elevated adipose-to-serum estrogen concentration ratios [36,37]. These findings suggest potential synergistic or interactive effects between endogenous adipose-derived estrogen and exogenous HT. The potential interaction between BMI and HT warrants further investigation in prospective studies. Although HT use was strongly associated with higher socioeconomic status (Table 1), HRQoL differences between HT users and non-users within each income stratum were not statistically significant (Table 2). This pattern suggests that the overall association between HT and higher EQ-5D scores may be partially attributable to the socioeconomic profile of HT users rather than the treatment itself, underscoring the importance of addressing socioeconomic confounding when interpreting cross-sectional findings.

**Figure 2:**
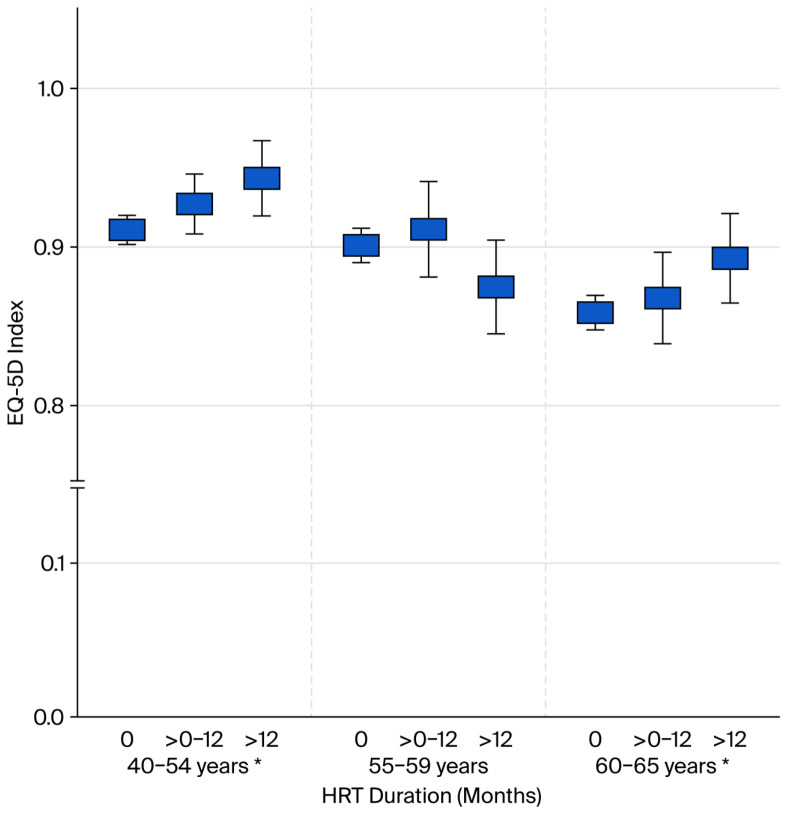
Mean EQ-5D index according to HT duration by age group. HT duration was classified into three categories: no HT use (0 months), >0–12 months, and >12 months. Error bars represent 95% CIs. * *p* for trend < 0.05.

It is further illustrated in Figure 2 that EQ-5D scores increased with longer HT duration, particularly among women aged <55 years and 60–65 years (*p* for trend < 0.05), consistent with a possible duration-dependent association and the relevance of treatment timing and persistence. The absence of a similar trend in the 55–59-year age group may reflect a transitional period during which symptom patterns and treatment response differ, although this finding warrants further study. Strengths of this study include the nationally representative sample drawn from the KNHANES; the use of a validated HRQoL instrument (EQ-5D-3L) with Korean-specific value weights; comprehensive subgroup analyses across age, BMI, education, and income strata; and the examination of both dimension-specific associations and duration-related trends.

Despite the above study strengths, several limitations should be acknowledged. First, the cross-sectional design precludes causal inference; observed associations may reflect selection bias (i.e., healthier women being more likely to use HT) rather than treatment effects. Second, self-reported HT use may be subject to recall bias, and the lack of detailed information on HT formulation, dosage, and route of administration limits assessment of dose–response relationships or comparisons across regimens. In South Korea during the study period (2005–2009), the most commonly prescribed HT formulations included estrogen therapy (ET) and combined estrogen–progestogen therapy (EPT), with usage patterns varying by demographic factors [38]. Unfortunately, the KNHANES dataset does not include specific information on HT formulation, dose, or route of administration, thus representing a limitation of this analysis. Third, residual confounding by indication remains possible because women who use HT may differ systematically from non-users in unmeasured factors such as symptom severity, health awareness, or comorbidity burden. The differential HRQoL profiles between current and past users also warrant consideration. Longitudinal studies are needed to characterize the trajectory of HRQoL changes following HT cessation. Fourth, although widely used, the EQ-5D-3L may have limited sensitivity to detect small but clinically meaningful differences because of ceiling effects in relatively healthy populations. Finally, we acknowledge that our data were collected between 2005 and 2009, which represents a limitation of this study; however, these years were specifically selected because they represent the only KNHANES cycles in which comprehensive HRQoL assessment using the EQ-5D instrument was collected concurrently with detailed HT usage data among postmenopausal women. While HT prescribing patterns and formulations may have evolved since this period, the fundamental physiological relationship between estrogen supplementation and quality-of-life dimensions remains biologically relevant. Moreover, these data provide important historical context for understanding long-term trends in HT outcomes in Asian populations, where longitudinal evidence remains limited.

While the cross-sectional design precludes causal inference and the data reflect HT practices from 2005 to 2009, our findings remain valuable for several key reasons: (1) the biological mechanisms linking estrogen to quality-of-life domains are well-established and temporally stable; (2) this research represents one of the few population-based studies examining HRQoL in Asian postmenopausal women using a validated instrument; and (3) the large, nationally representative sample provides robust statistical power. Nevertheless, we acknowledge that contemporary HT formulations, dosing strategies, and sample selection criteria may differ from the study period, and caution should be exercised when extrapolating these findings to current clinical practice. Prospective longitudinal studies with contemporary data are required to establish temporal relationships and confirm these associations.

## 5. Conclusions

The findings of this nationwide cross-sectional study demonstrate significant associations between HT and HRQoL among postmenopausal Korean women. HT use was associated with higher overall EQ-5D index scores, with significant differences observed between women aged <55 and ≥60 years. The EQ-5D index was positively associated with HT duration in younger and older age groups, supporting a potential duration-dependent relationship. In age-adjusted analyses, HT use was associated with lower odds of reporting problems across all five EQ-5D dimensions, with the strongest association observed for usual activities. Among BMI subgroups, the HRQoL difference favoring HT users was statistically significant only among women with a BMI of ≥25.0 kg/m^2^. These findings support individualized menopausal counseling that integrates quality-of-life considerations with established risk–benefit assessments. Future longitudinal studies with detailed information on HT regimens are required to clarify causal relationships and to identify the subgroups most likely to derive meaningful HRQoL benefit from HT.

## Figures and Tables

**Table 1:** Baseline characteristics of study participants by hormone therapy (HT) status.

Characteristics	*N*	HT Users (%)	*p*-Value ^1^
Total	2460	464 (18.9)	
Age (years)			0.082
<55	803	164 (20.4)	
55–59	742	143 (19.3)	
≥60	915	157 (17.2)	
BMI (kg/m^2^)			0.010
<18.5	43	5 (11.6)	
18.5–22.9	754	171 (22.7)	
23.0–24.9	674	123 (18.3)	
≥25.0	989	165 (16.7)	
Education			<0.001
≤Elementary school	1359	199 (14.6)	
Middle school	487	106 (21.8)	
≥High school	614	159 (25.9)	
Income			<0.001
Quartile 1 (lowest)	620	92 (14.8)	
Quartile 2	622	100 (16.1)	
Quartile 3	622	121 (19.5)	
Quartile 4 (highest)	596	151 (25.3)	

^1^ *p*-values were determined using the Mantel–Haenszel chi-square test.

**Table 2:** Mean EQ-5D index by demographic characteristics stratified by hormone therapy (HT) status.

Characteristics	All	Non-HT Users (*n* = 1996)	HT Users (*n* = 464)
Total	0.898 (0.893–0.903)	0.894 (0.889–0.900)	0.911 (0.902–0.921) *
Age (years)			
<55	0.923 (0.916–0.930)	0.919 (0.911–0.927)	0.939 (0.925–0.952) *
55–59	0.903 (0.894–0.912)	0.906 (0.896–0.917)	0.892 (0.873–0.910)
≥60	0.863 (0.854–0.873)	0.857 (0.846–0.868)	0.895 (0.877–0.913) *
BMI (kg/m^2^)			
<18.5	0.902 (0.867–0.937)	0.898 (0.859–0.938)	0.919 (0.830–1.008)
18.5–22.9	0.917 (0.908–0.925)	0.913 (0.902–0.923)	0.929 (0.915–0.944)
23.0–24.9	0.908 (0.899–0.916)	0.909 (0.899–0.919)	0.901 (0.882–0.921)
≥25.0	0.876 (0.867–0.885)	0.871 (0.861–0.881)	0.900 (0.882–0.918) *
Education			
≤Elementary school	0.873 (0.865–0.881)	0.872 (0.864–0.881)	0.876 (0.860–0.893)
Middle school	0.910 (0.899–0.920)	0.904 (0.892–0.916)	0.927 (0.906–0.949)
≥High school	0.936 (0.929–0.943)	0.935 (0.926–0.943)	0.938 (0.926–0.950)
Income			
Quartile 1 (lowest)	0.873 (0.861–0.884)	0.869 (0.856–0.881)	0.892 (0.866–0.918)
Quartile 2	0.892 (0.882–0.902)	0.892 (0.881–0.903)	0.894 (0.873–0.914)
Quartile 3	0.903 (0.893–0.913)	0.901 (0.889–0.912)	0.913 (0.895–0.931)
Quartile 4 (highest)	0.922 (0.913–0.931)	0.917 (0.906–0.928)	0.935 (0.921–0.950)

Data are presented as weighted mean (95% confidence interval). * *p* < 0.05 between non-HT and HT users by weighted *t*-tests.

**Table 3:** Age-adjusted odds ratios (95% CI) for reporting problems in each EQ-5D dimension by hormone therapy (HT) status.

EQ-5D Dimension	Non-HT Users (*n* = 1996)	HT Users (*n* = 464)	*p*
Mobility	1.000 (reference)	0.869 (0.865–0.872)	<0.001
Self-care	1.000 (reference)	0.855 (0.848–0.862)	<0.001
Usual activities	1.000 (reference)	0.719 (0.715–0.722)	<0.001
Pain/discomfort	1.000 (reference)	0.990 (0.987–0.993)	<0.001
Anxiety/depression	1.000 (reference)	0.915 (0.911–0.918)	<0.001

**Table 4:** Age-stratified fully adjusted odds ratios (95% CI) for reporting problems in each EQ-5D dimension by hormone therapy (HT) status.

Age Group	Non-HT Users	HT Users	*p*
40–54 years			
Mobility	1.000 (reference)	0.961 (0.954–0.969)	<0.001
Self-care	1.000 (reference)	0.574 (0.562–0.586)	<0.001
Usual activities	1.000 (reference)	0.547 (0.541–0.553)	<0.001
Pain/discomfort	1.000 (reference)	0.981 (0.976–0.986)	<0.001
Anxiety/depression	1.000 (reference)	0.780 (0.775–0.785)	<0.001
55–59 years			
Mobility	1.000 (reference)	1.660 (1.650–1.670)	<0.001
Self-care	1.000 (reference)	1.859 (1.837–1.883)	<0.001
Usual activities	1.000 (reference)	1.161 (1.153–1.170)	<0.001
Pain/discomfort	1.000 (reference)	1.394 (1.387–1.402)	<0.001
Anxiety/depression	1.000 (reference)	1.338 (1.329–1.347)	<0.001
60–65 years			
Mobility	1.000 (reference)	0.707 (0.702–0.712)	<0.001
Self-care	1.000 (reference)	0.620 (0.611–0.630)	<0.001
Usual activities	1.000 (reference)	0.868 (0.861–0.875)	<0.001
Pain/discomfort	1.000 (reference)	0.967 (0.961–0.973)	<0.001
Anxiety/depression	1.000 (reference)	1.005 (0.998–1.012)	0.174

Adjusted for body mass index, education level, and household income.

## Data Availability

The data used to support the findings of this study are available from the corresponding author upon request.
